# Oral DNA Demethylating Agents and Epigenetic Targeting in Adult T-Cell Leukemia/Lymphoma

**DOI:** 10.32604/or.2026.084740

**Published:** 2026-10-28

**Authors:** Yuta Yamamoto, Hiroshi Ureshino, Shinya Kimura

**Affiliations:** 1 Division of Hematology, Respiratory Medicine and Oncology, Department of Internal Medicine, Faculty of Medicine, Saga University, Saga, Japan; 2 Department of Drug Discovery and Biomedical Sciences, Faculty of Medicine, Saga University, Saga, Japan

**Keywords:** DNA methylation, DNA demethylating agents, epigenetic therapy, OR-2100, adult T-cell leukemia/lymphoma

## Abstract

DNA methylation plays a critical role in gene regulation and is frequently dysregulated in hematological malignancies such as myelodysplastic syndromes and acute myeloid leukemia. DNA demethylating agents have therefore emerged as important therapeutic options. However, conventional DNA demethylating agents require parenteral administration, which limits their long-term use and patient convenience. In this review, we aim to provide an overview of recent advances in the development of orally available DNA demethylating agents and discuss their therapeutic applications in hematological malignancies. Orally available DNA demethylating agents, such as CC-486 (oral azacitidine) and ASTX727 (a fixed-dose combination of oral decitabine and the cytidine deaminase inhibitor cedazuridine), have been developed to overcome these limitations and enable sustained drug exposure. In addition, OR-2100, a novel oral prodrug of decitabine, has been shown to exhibit improved pharmacokinetic properties and reduced hematologic toxicity in preclinical studies. These findings suggest that orally available DNA demethylating agents may expand therapeutic options, particularly for hematological malignancies such as adult T-cell leukemia/lymphoma.

## 1 Introduction

Multiple factors contribute to human carcinogenesis, with approximately 12%–20% of cancers attributed to viral infections [1]. Established oncogenic viruses include the Epstein–Barr virus, human papillomavirus, and hepatitis viruses [1]. This review focuses on adult T-cell leukemia/lymphoma (ATL), a malignancy caused by human T-cell lymphotropic virus type-1 (HTLV-1). ATL is endemic in southwestern Japan, particularly in the Kyushu region, and remains a significant regional health concern [2,3,4]. ATL is a peripheral T-cell malignancy that develops when HTLV-1-infected T cells undergo malignant transformation and acquire clonal proliferative capacity [5,6]. Following a prolonged latency period spanning several decades, infected cells accumulate genetic and epigenetic alterations, ultimately leading to the clinical onset of ATL [7,8]. Despite widespread infection, only a small proportion of carriers develop ATL, with an estimated annual incidence of 1–2 cases per 1000 infected individuals [3,9]. Clinically, ATL is classified into four subtypes—smoldering, chronic, acute, and lymphoma—based on disease presentation [6,9]. Smoldering ATL and chronic ATL without adverse prognostic factors are considered indolent and are typically managed using a watchful waiting approach [6,9]. In contrast, acute and lymphoma subtypes as well as chronic ATL with poor prognostic features, are categorized as aggressive disease and are associated with unfavorable clinical outcomes [10]. These aggressive forms generally require intensive combination chemotherapy, often combined with antiviral therapy or allogeneic hematopoietic stem cell transplantation [6,9,10].

Accumulating evidence highlights the critical role of epigenetic dysregulation, particularly aberrant DNA methylation, in the multistep pathogenesis of ATL [7,8,11,12]. DNA methylation abnormalities are increasingly recognized as key drivers of transcriptional dysregulation and malignant phenotypes in hematologic malignancies, including ATL. Consequently, epigenetic therapy has emerged as a promising treatment strategy, and DNA demethylating agents have garnered considerable interest in both preclinical and clinical settings. This review aims to summarize current insights into DNA methylation abnormalities in ATL oncogenesis and to discuss recent advances in DNA demethylating therapies, particularly focusing on orally available agents.

## 2 DNA Methylation

In mammals, DNA methylation primarily involves the addition of a methyl group (CH_3_) to the 5-carbon position of the cytosine pyrimidine ring, a reaction catalyzed by DNA methyltransferases DNMT1, DNMT3A, and DNMT3B [13]. S-adenosyl-L-methionine functions as the methyl donor in this process. DNMT1 is responsible for maintenance methylation, whereby methylation patterns are preserved during DNA replication through modification of newly synthesized daughter strands. In contrast, DNMT3A and DNMT3B mediate *de novo* methylation at previously unmethylated CpG sites [13]. In the mammalian genome, approximately 60%–90% of CpG cytosines are methylated, contributing to the regulation of gene expression and maintenance of chromosomal stability [13]. CpG islands, which are typically located near transcription start sites, are generally unmethylated in transcriptionally active genes; however, methylation of these regions is strongly associated with transcriptional silencing [13]. Genome-wide analyses have further demonstrated that the functional consequences of DNA methylation depend on the genomic context of CpG sites [13]. Accordingly, aberrant DNA methylation can alter cellular phenotypes and has been implicated in a broad spectrum of diseases, including cancer, lifestyle-related disorders, and psychiatric conditions.

## 3 DNA Hypermethylation in ATL Oncogenesis

ATL develops through a multistep carcinogenic process in which HTLV-1-infected T cells progressively accumulate genetic and epigenetic alterations over several decades [5]. To elucidate the role of DNA methylation in ATL, we conducted comprehensive methylation analyses [11]. Because DNA methylation patterns are influenced by aging and lifestyle factors [14,15], these variables were considered in the analytical framework. Normal T cells and HTLV-1-infected T cells (ATL cells) were isolated from peripheral blood mononuclear cells (PBMCs) obtained from healthy individuals, asymptomatic carriers, and patients with ATL using the HTLV-1 analysis system by flow cytometry (Has-Flow) [16]. This approach identifies HTLV-1-infected cells based on the expression of CD3, CD4, CD7, and cell adhesion molecule 1 (CADM1) (Fig. 1A). CADM1 is a cell adhesion molecule upregulated in HTLV-1-infected cells, whereas CD7 expression progressively decreases during ATL progression [17,18]. In addition to serving as a phenotypic marker, CADM1 (TSLC1/IGSF4) may contribute to cell–cell interaction, tissue infiltration, and activation signaling pathways such as NF-κB. In contrast, CD7 is a pan–T-cell marker, and its progressive downregulation has been associated with altered T-cell signaling and immune evasion [16,18]. Therefore, the characteristic CADM1 upregulation and CD7 downregulation in HTLV-1–infected cells are considered not merely as biomarkers but also as features reflecting biological changes associated with clonal expansion and disease progression. The Has-Flow method enables precise discrimination between normal and HTLV-1-infected T-cell populations. Specifically, healthy CD3^+^CD4^+^ T cells are predominantly detected in the P fraction (CADM1^−^CD7^+^), whereas HTLV-1-infected cells are distributed across the D (CADM1^+^CD7^+^) and N (CADM1^+^CD7^−^) fractions. Notably, the N fraction predominates in acute ATL (Fig. 1B). Genome-wide methylation profiling was performed using Infinium HumanMethylation450 or Illumina Infinium MethylationEPIC BeadChip arrays [11]. Hierarchical clustering analysis revealed that all P fractions clustered together irrespective of infection status, whereas the D and N fractions exhibited distinct methylation patterns characterized by relative hypomethylation. Furthermore, the N fraction formed a separate cluster in acute ATL (Fig. 1C). Differentially methylated positions (DMPs) were subsequently identified. Hypermethylated DMPs showed a strong correlation with disease progression, whereas hypomethylated DMPs did not demonstrate such an association (Fig. 1D,E). Collectively, these findings indicate that DNA hypermethylation plays a critical role in the development and progression of ATL. In addition to these findings, accumulating evidence indicates that ATL is characterized by widespread epigenetic alterations affecting the regulation of both oncogenes and tumor suppressor genes [5,19].

**Figure 1:**
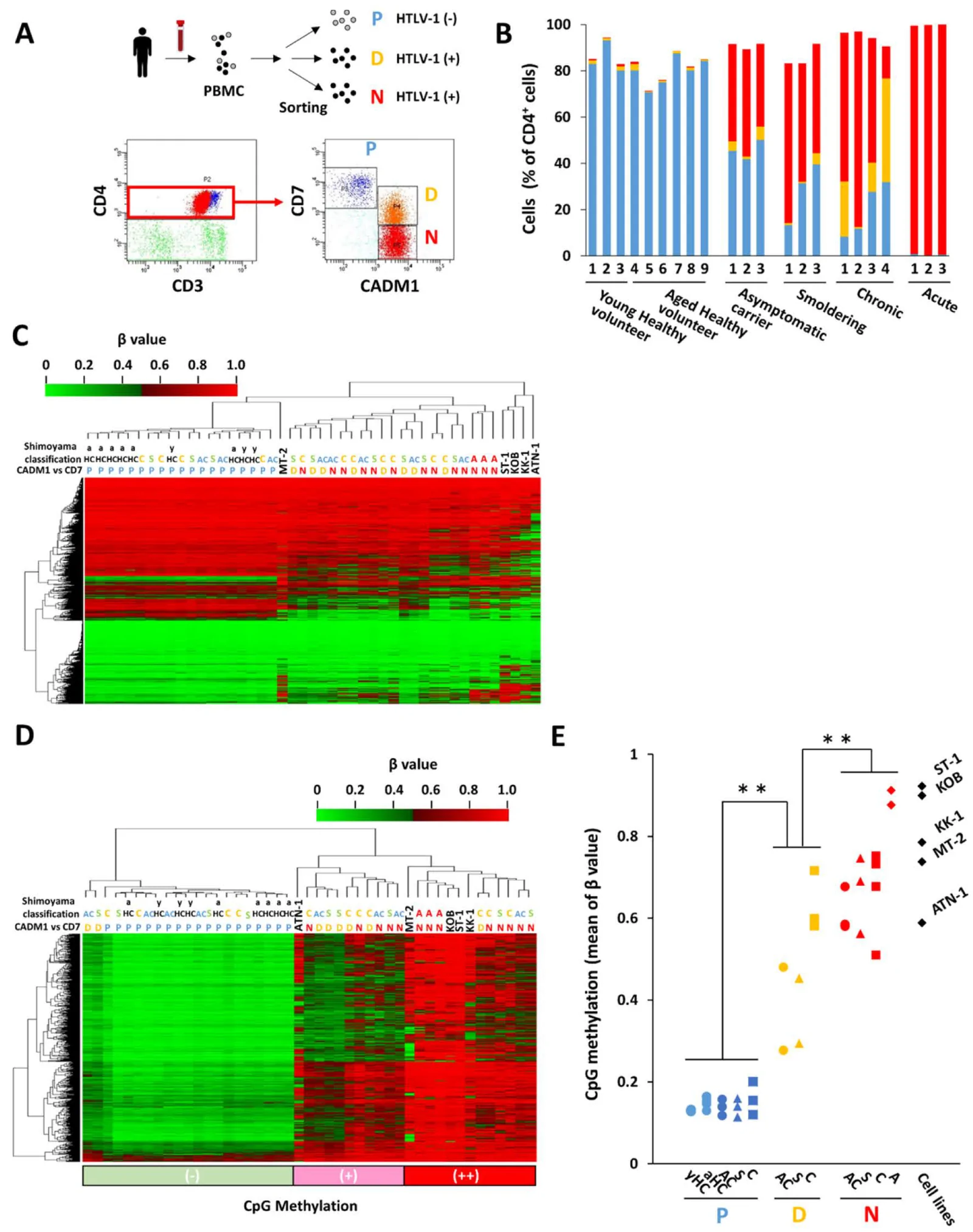
**Progressive DNA hypermethylation during ATL development and disease progression.** (**A**) HTLV-1–infected cells and uninfected normal T cells were separated based on CADM1 and CD7 expression using the Has-Flow method. (**B**) The proportions of P (blue), D (yellow), and N (red) fractions in samples used for genome-wide DNA methylation analysis are shown. (**C**) Heatmap of hierarchical clustering based on methylation profiles of 20,000 randomly selected CpG sites across the genome; red indicates methylated CpG sites and green indicates unmethylated CpG sites. (**D**) Hierarchical clustering heatmap showing ATL-specific hypermethylation in HTLV-1–infected cells. (**E**) Average DNA methylation levels (β values) of the CpG sites shown in panel D. ATL: adult T-cell leukemia/lymphoma; HTLV-1: human T-cell lymphotropic virus type-1; CADM1: cell adhesion molecule 1. Adapted from [11]. **p < 0.01. Copyright © 2020 American Society of Hematology.

## 4 DNA Demethylating Agents

### 4.1 Development of Azacitidine and Decitabine

Aberrant DNA methylation in cancer, particularly promoter hypermethylation of tumor suppressor genes such as RB, p16, p14ARF, VHL, and hMLH1, has long been recognized as a therapeutic target. Reversal of these epigenetic alterations can restore gene expression and suppress malignant phenotypes [20,21]. More recently, promoter hypermethylation has also been identified in a wider range of tumor suppressor genes, microRNAs, and long non-coding RNAs, thereby expanding both preclinical and clinical interest in DNA demethylating agents. Among representative agents, azacitidine—currently approved for clinical use in Japan and other countries—was originally synthesized in 1964 as a nucleic acid synthesis inhibitor [22]. Although it demonstrated antimetabolite activity and growth-inhibitory effects in leukemia cell lines, it was not initially developed as a conventional anticancer drug. In the 1980s, however, azacitidine was found to inhibit DNA methylation and promote cellular differentiation [23]. At that time, aberrant DNA hypermethylation had been implicated in the pathogenesis of myelodysplastic syndromes (MDS), which led to the repositioning of azacitidine as an epigenetic therapy. This ultimately resulted in its approval by the U.S. Food and Drug Administration (FDA) in 2004. Decitabine, which is also approved in the United States and Europe, was first synthesized in 1964 and later demonstrated antitumor activity in a virus-associated leukemogenesis model using AKR/J (AKR) mice in 1968 [24]. Similar to azacitidine, decitabine was subsequently shown to possess DNA demethylating and differentiation-inducing properties, leading to its FDA approval in 2006 [23].

### 4.2 Mechanisms and Biological Effects of Azacitidine and Decitabine

Following cellular uptake via nucleoside transporters, azacitidine and decitabine undergo sequential phosphorylation and are incorporated into RNA and DNA. Due to its chemical structure, approximately 80%–90% of intracellular azacitidine is incorporated into RNA, while 10%–20% is converted by ribonucleotide reductase into its deoxyribonucleotide form and incorporated into DNA [25]. During DNA replication, azacytosine incorporated into DNA is recognized by DNMT1 in a manner similar to cytosine. However, because the 5-position of the pyrimidine ring is replaced by a nitrogen atom, the methyl transfer reaction cannot be completed. As a result, DNMT1 becomes covalently trapped on DNA and is subsequently degraded via the ubiquitin–proteasome pathway. Through this mechanism, azacitidine and decitabine induce cell cycle-dependent DNA demethylation [26]. In addition to their classical mechanisms of action, recent studies have highlighted that DNA demethylating agents can modulate the tumor immune microenvironment through the induction of immunogenic cell death (ICD). Demethylation-mediated reactivation of endogenous retroviral elements leads to the accumulation of double-stranded RNA, thereby triggering type I interferon responses and enhancing tumor immunogenicity [27,28]. Furthermore, these agents have been shown to influence the expression of non-coding RNAs, including microRNAs and long non-coding RNAs, which contribute to the regulation of oncogenic pathways and tumor suppressor gene expression [29,30]. These findings suggest that the biological effects of DNA demethylating agents extend beyond direct cytotoxicity and epigenetic reprogramming to include broader immunological and transcriptional regulation. Because the epigenetic effects of DNA demethylating agents are dependent on DNA replication and require sustained exposure, treatment regimens typically involve consecutive multi-day administration. However, in clinical practice, the need for intravenous or subcutaneous administration of azacitidine and decitabine imposes a considerable burden on patients and limits long-term treatment flexibility. In addition, cumulative hematologic toxicity further restricts their use. These limitations have driven efforts to optimize pharmacokinetic properties and delivery strategies, ultimately leading to the development of orally available DNA demethylating agents.

## 5 Development of Oral DNA Demethylating Agents

### 5.1 CC-486 (Oral Azacitidine, ONUREG^®^)

Although azacitidine and decitabine have become standard therapies for myeloid malignancies, both require daily intravenous or subcutaneous administration. This regimen imposes a substantial burden on patients and limits long-term treatment flexibility [31]. To address these limitations, CC-486 was developed as an orally bioavailable formulation of azacitidine, enabling prolonged dosing schedules suitable for maintenance and long-term therapy [31,32,33]. Early clinical studies in patients with MDS, chronic myelomonocytic leukemia (CMML), and acute myeloid leukemia (AML) demonstrated that CC-486 achieves systemic exposure sufficient to induce DNA demethylation without significant drug accumulation during repeated dosing [34]. Pharmacodynamic analyses further indicated that extended oral administration results in sustained DNA demethylation, with the degree of demethylation correlating with systemic drug exposure and hematologic responses [34].

The clinical efficacy of this strategy was subsequently confirmed in the QUAZAR AML-001 trial, a randomized phase III study evaluating CC-486 as maintenance therapy in patients with AML who achieved complete remission but were ineligible for hematopoietic stem cell transplantation [35]. CC-486 significantly improved overall survival and relapse-free survival compared with placebo while maintaining acceptable health-related quality of life during long-term administration [35]. Adverse events were observed, predominantly gastrointestinal and hematologic toxicities; however, these were generally manageable in the maintenance setting [35]. Based on these findings, CC-486 was approved by the U.S. FDA in 2020 for post-remission maintenance therapy in AML.

### 5.2 ASTX727 (Oral Decitabine/Cedazuridine, INQOVI^®^)

Decitabine is rapidly degraded by cytidine deaminase (CDA) in the gastrointestinal tract and liver, which has historically restricted its clinical use to parenteral administration [36]. ASTX727 was developed as a fixed-dose combination of decitabine and cedazuridine, a potent CDA inhibitor, to enable oral delivery by inhibiting metabolic degradation of decitabine [33,36]. Early clinical studies demonstrated that oral ASTX727 achieved systemic exposure comparable to that of intravenous decitabine, as assessed by the area under the concentration–time curve, while preserving DNA demethylating activity [37].

Pharmacokinetic and pharmacodynamic equivalence between oral ASTX727 and intravenous decitabine was further confirmed in later-phase trials, establishing that pharmacological inhibition of CDA could effectively compensate for the metabolic instability of decitabine [37]. The adverse event profile of ASTX727 was largely comparable to that of intravenous decitabine, with hematologic toxicity representing the most common treatment-related adverse event [38]. Based on these clinical findings, ASTX727 was approved by the U.S. FDA in 2020 for the treatment of patients with high-risk MDS and CMML.

### 5.3 OR-2100 (Oral Decitabine Prodrug)

Collectively, CC-486 and ASTX727 represent two distinct strategies for improving the clinical feasibility of DNA demethylating therapy—namely, optimization of dosing schedules and pharmacological inhibition of metabolic degradation [34,36]. These developments provided an important foundation for oral administration; however, they also underscored the limitations of approaches that depend on external compensatory mechanisms. Although these agents constitute significant advances, their long-term use still relies on external strategies to compensate for metabolic instability, highlighting the need for alternative approaches that inherently integrate oral bioavailability, sustained systemic exposure, and tolerability within a single molecular framework. In contrast, strategies based on intrinsic molecular design aim to overcome these limitations at the pharmacokinetic level rather than through external compensation.

In this context, we sought to develop a single-molecule oral prodrug of decitabine designed to confer intrinsic resistance to CDA-mediated degradation while retaining epigenetic activity [39,40]. To illustrate the structural basis of this design concept, the chemical structures of decitabine and its oral prodrug OR-2100 are shown in Fig. 2A,B. A series of 5′-O-trialkylsilylated decitabine derivatives were synthesized and systematically evaluated. DNA demethylating activity was first assessed using a luciferase-based reporter assay sensitive to promoter hypermethylation [41] and subsequently validated by quantitative analysis of LINE-1 methylation as a surrogate marker of global genomic DNA methylation [40]. OR-2100 is a prodrug of decitabine that becomes biologically active through the release of DAC following the cleavage of its 5′-O-trialkylsilyl moiety. The selection of OR-2100 as the lead compound was based on an integrated assessment of lipophilicity, metabolic stability, and controlled decitabine release. OR-2100 exhibited a logP value compatible with oral absorption and demonstrated marked resistance to CDA-mediated degradation *in vitro*, in contrast to the rapid degradation observed with decitabine [39]. Pharmacokinetic analyses further confirmed the improved bioavailability of OR-2100. Following duodenal administration in cynomolgus monkeys, OR-2100 achieved substantially higher systemic exposure than decitabine when administered at comparable doses, reflecting its higher intestinal bioavailability. Notably, systemic exposure was markedly greater, approximately 30-fold higher than that achieved with decitabine alone under comparable conditions [11]. Consistent with these findings, OR-2100 administration induced DNA hypomethylation PBMCs, including at the proximal promoter region of the γ-globin gene [40]. Genome-wide analyses revealed highly overlapping DNA methylation and transcriptional profiles between OR-2100 and decitabine, indicating that the epigenetic effects of OR-2100 are mediated through controlled release of decitabine [39]. In multiple mouse models, OR-2100 retained antitumor efficacy while exhibiting reduced hematologic toxicity compared with decitabine [39,40]. This favorable profile is consistent with a broader concentration–time exposure pattern that supports non-cytotoxic depletion of DNMT1 [42]. Collectively, these findings indicate that OR-2100 enables safe and sustained epigenetic modulation through a distinct pharmacokinetic profile derived from rational prodrug design, supporting its potential utility for long-term oral administration. Importantly, although the development of OR-2100 was driven by pharmacokinetic considerations and oral bioavailability, its therapeutic potential must ultimately be evaluated in disease-specific contexts characterized by pronounced epigenetic abnormalities.

**Figure 2:**
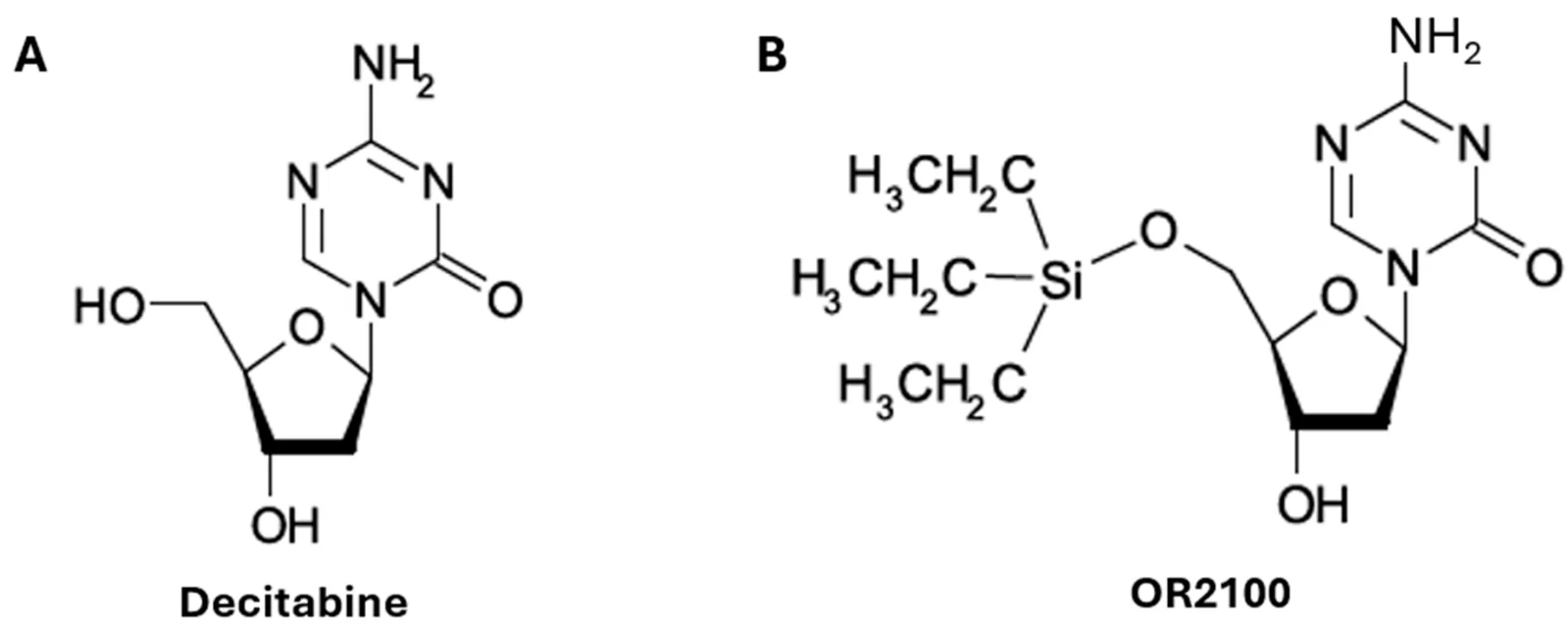
**Chemical structures of decitabine and OR-2100.** (**A**) Chemical structure of decitabine. (**B**) Chemical structure of OR-2100, a silylated prodrug of decitabine. Adapted from [40]. Copyright © 2019 American Association for Cancer Research.

## 6 Anti-ATL Therapeutic Effects of OR-2100

Although several DNA demethylating agents have demonstrated clinical efficacy in hematological malignancies such as AML, their application in ATL remains extremely limited, likely reflecting differences in disease Given these limitations, OR-2100 has emerged as a novel oral DNA demethylating agent with potential therapeutic relevance in ATL. Based on our previous findings that characteristic DNA hypermethylation progressively accumulates in HTLV-1–infected cells during the development and progression of ATL, we evaluated the therapeutic efficacy of DNA demethylating agents in ATL [11]. *In vitro* analyses showed that the established agents decitabine and azacitidine suppressed the proliferation of HTLV-1–infected cell lines in a concentration-dependent manner and significantly reduced methylation in the proximal promoter region of the LINE-1 gene, a surrogate marker of global DNA methylation [11]. Under these experimental conditions, OR-2100 exhibited DNA demethylating activity and anti-ATL effects comparable to those of decitabine [11]. These findings provided a rationale for further evaluation of OR-2100 in *in vivo* models, where its antitumor efficacy was subsequently confirmed. In xenograft models using HTLV-1–infected cell lines, OR-2100 suppressed tumor growth and induced DNA demethylation to a similar extent as decitabine; however, OR-2100 caused a smaller reduction in red blood cells and neutrophils, suggesting lower hematologic toxicity (Fig. 3A,B). Furthermore, in a patient-derived xenograft (PDX) mouse model established using PBMCs from patients with chronic-type ATL, OR-2100 treatment significantly prolonged survival compared with the control group (Fig. 3C).

**Figure 3:**
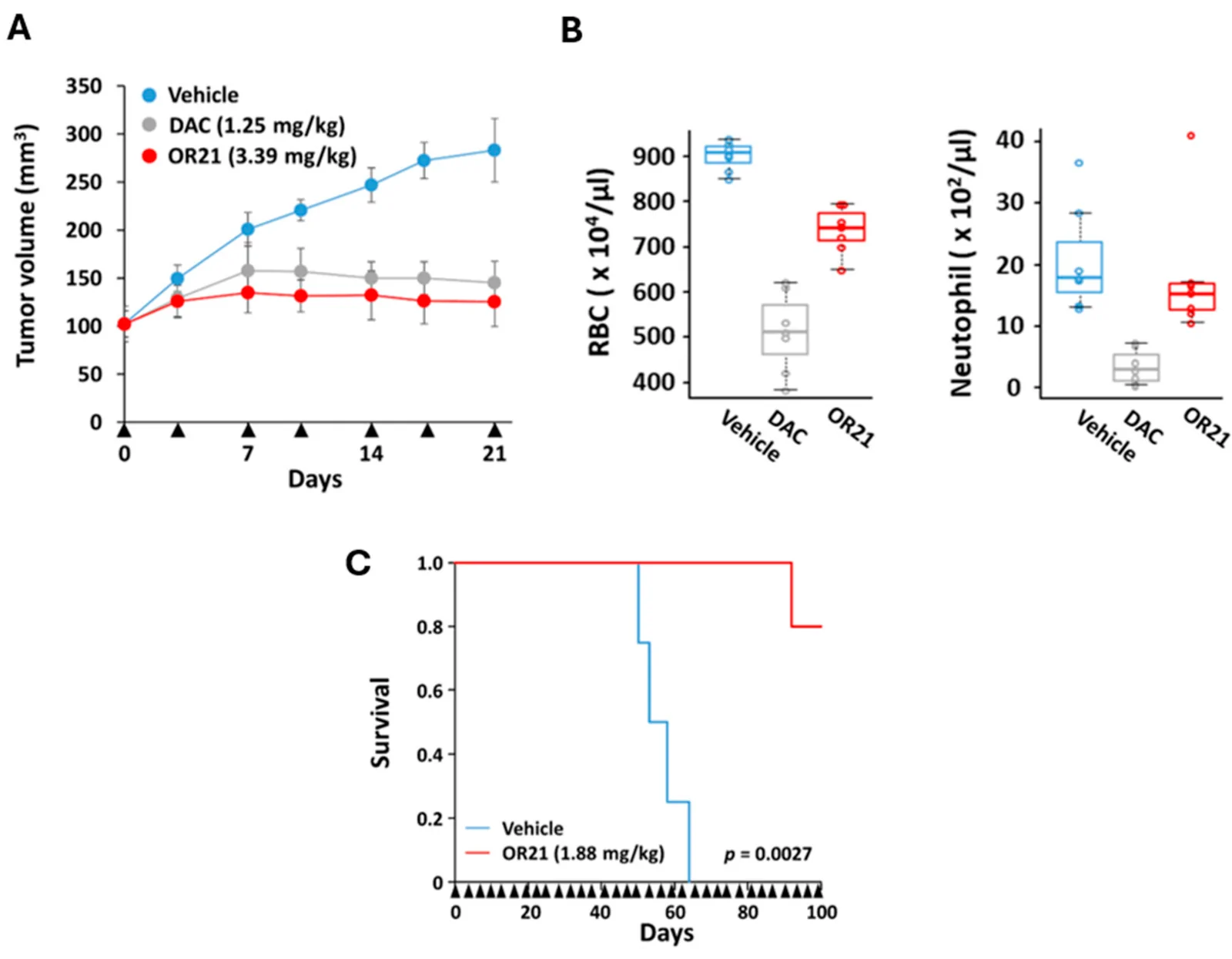
**Anti-ATL effects of the novel DNA demethylating agent OR-2100.** (**A**) After transplantation of the HTLV-1–infected MT-2 cell line into severely immunodeficient BRJ mice, treatment was initiated after tumor establishment, approximately 11 days after transplantation, when the average tumor volume reached approximately 100 mm^3^. Decitabine, OR-2100 adjusted to achieve equivalent systemic exposure (AUC) of released decitabine, or vehicle was administered intraperitoneally twice weekly, and tumor growth was monitored over time. (**B**) Peripheral blood cell counts at the time of sacrifice are shown. (**C**) Peripheral blood mononuclear cells isolated from patients with chronic-type ATL were transplanted into BRJ mice, followed by intraperitoneal administration of OR-2100 twice weekly, and overall survival was evaluated. ATL: adult T-cell leukemia/lymphoma; HTLV-1: human T-cell lymphotropic virus type-1; BRJ: BALB/c, Rag-2-/-/Jak3-/-; Adapted from [11]. Copyright © 2020 American Society of Hematology.

## 7 Epigenetic Chemoprevention Using OR-2100 in a Virus-Associated Leukemia Model

In addition to its therapeutic efficacy, we hypothesized that sustained modulation of aberrant DNA methylation may prevent the progression of virus-associated leukemogenesis. Because no genetically engineered or spontaneous mouse model that fully recapitulates ATL is currently available, we used AKR mice as an established model of retrovirus-associated lymphoid leukemia [43]. AKR mice spontaneously develop lymphoid leukemia after a long latency period, with disease onset occurring at approximately 200 days of age and nearly all animals succumbing to leukemia by around 300 days [44]. Young AKR mice were treated with OR-2100 or decitabine at dose levels adjusted to achieve equivalent systemic exposure, as determined by area under the concentration–time curve (AUC). Untreated control mice developed leukemia and died at approximately 300 days of age. In contrast, mice treated with decitabine died earlier without overt leukemia, likely due to cumulative hematologic toxicity, including anemia. At high dose levels, both OR-2100 and decitabine induced hematologic toxicity; however, differences in the severity of toxicity were observed even at comparable systemic exposure levels. Notably, OR-2100-treated mice exhibited significantly prolonged survival, with a subset surviving beyond 500 days (Fig. 4A) [45]. DNA methylation analysis of thymic tumor cells isolated from AKR mice revealed a pattern of global hypomethylation accompanied by focal promoter hypermethylation, closely resembling the epigenetic features observed in ATL cells (Fig. 4B). In addition, thymic tumor cells from AKR mice showed marked overexpression of MYC protein (Fig. 4C). *In vitro* analyses using AKR-derived tumor cell lines further demonstrated that OR-2100 suppressed cellular proliferation and induced demethylation of repetitive elements such as LINE-1 (Fig. 4D,E). Furthermore, OR-2100 treatment resulted in a dose-dependent reduction in MYC protein expression in AKR-derived tumor cell lines (Fig. 4F). These findings suggest that MYC overexpression may contribute to leukemogenic processes in this model and that sustained epigenetic modulation by OR-2100 may suppress oncogenic transcriptional programs associated with disease development. Collectively, these results indicate that DNA methylation abnormalities play a role in retrovirus-associated lymphoid leukemogenesis and suggest that OR-2100 may represent a promising epigenetic chemopreventive agent.

**Figure 4:**
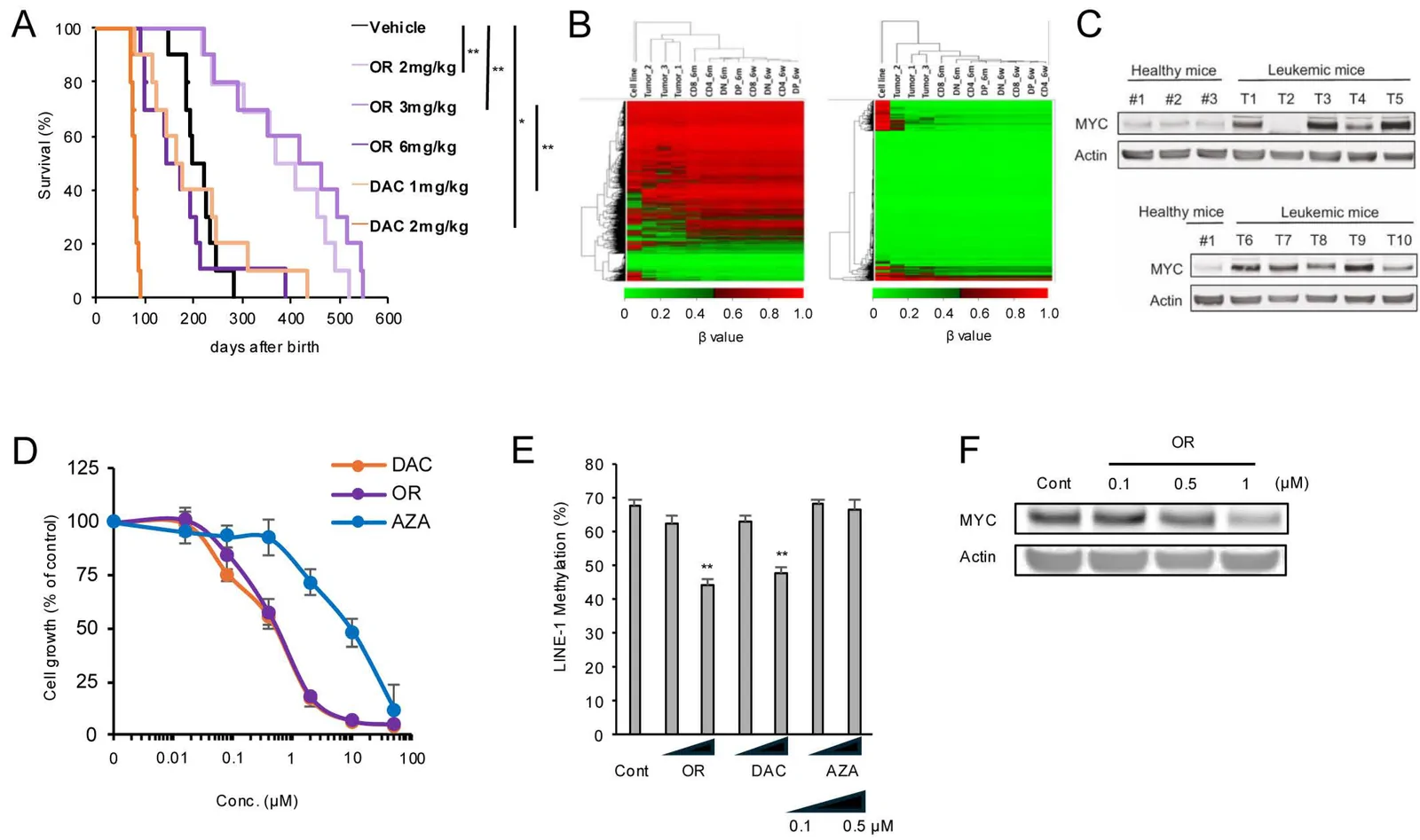
**Chemopreventive effects of OR-2100 in AKR mice.** (**A**) Survival curves of AKR mice treated intraperitoneally with OR-2100 or decitabine at doses adjusted to achieve equivalent systemic exposure (AUC). Pharmacokinetic analyses indicated that 3 mg/kg and 6 mg/kg OR-2100 produced AUCs of released decitabine comparable to those observed with 1 mg/kg and 2 mg/kg decitabine, respectively. Treatment was initiated at 6 weeks of age and administered intraperitoneally twice weekly until mice reached predefined experimental endpoints or death, *p < 0.05, **p < 0.01. (**B**) DNA methylation profiles of normal and tumor thymocytes from AKR mice based on randomly selected or promoter-associated CpG sites. (**C**) Representative immunoblot analysis of MYC protein expression in thymic tumor cells isolated from AKR mice. (**D**) Effects of 96-h treatment with DNA demethylating agents on AKR tumor cell lines, assessed by CCK-8 assay. (**E**) LINE-1 DNA methylation levels analyzed by bisulfite pyrosequencing following 96-h treatment. Statistical significance was determined relative to the untreated control using Dunnett’s test: **p < 0.01. (**F**) Immunoblot analysis of MYC protein expression in AKR tumor cells treated with OR-2100. AKR: AKR/J. AZA; azacitidine. Adapted from [45]. Copyright © 2024 Springer Nature Limited.

## 8 Conclusion

DNA demethylating agents have become an essential component of epigenetic therapy for hematological malignancies, as exemplified by the long-standing clinical use of azacitidine and decitabine. Advances in drug design have expanded their clinical utility by addressing key limitations associated with parenteral administration, leading to the successful development of orally available formulations such as CC-486 and ASTX727. These agents have demonstrated that sustained modulation of DNA methylation can be achieved in clinical settings; however, long-term treatment remains constrained by hematologic toxicity and limited therapeutic flexibility. OR-2100 represents a next-generation approach to epigenetic therapy based on rational molecular design. By incorporating resistance to CDA-mediated degradation within a single-molecule prodrug, OR-2100 enables oral administration while modifying the pharmacokinetic profile of decitabine toward prolonged, lower-intensity exposure. These advances highlight the potential of DNA demethylating agents, particularly in the context of ATL, as promising therapeutic options in hematological malignancies, with emerging implications for disease prevention.

## 9 Future Outlook

Preclinical studies suggest that this strategy may preserve anti-ATL therapeutic efficacy while reducing cumulative toxicity, potentially broadening the applicability of DNA demethylating therapy. In addition, long-term administration of OR-2100 in a retrovirus-associated leukemia model provides proof-of-concept evidence that sustained epigenetic modulation could influence not only established disease but also early stages of malignant progression. Although translation of epigenetic chemoprevention into clinical practice remains challenging and will require careful evaluation of the risk–benefit balance, these findings support the potential extension of epigenetic therapy beyond conventional treatment paradigms (Fig. 5). While epigenetic chemoprevention represents a promising concept, whether OR-2100 can induce sustained epigenetic changes in normal or early-infected cells remains to be fully elucidated. Currently, a phase I clinical trial of OR-2100 is ongoing in patients with MDS, and further clinical studies will be necessary to determine the optimal contexts in which next-generation oral DNA demethylating agents can be safely and effectively applied.

**Figure 5:**
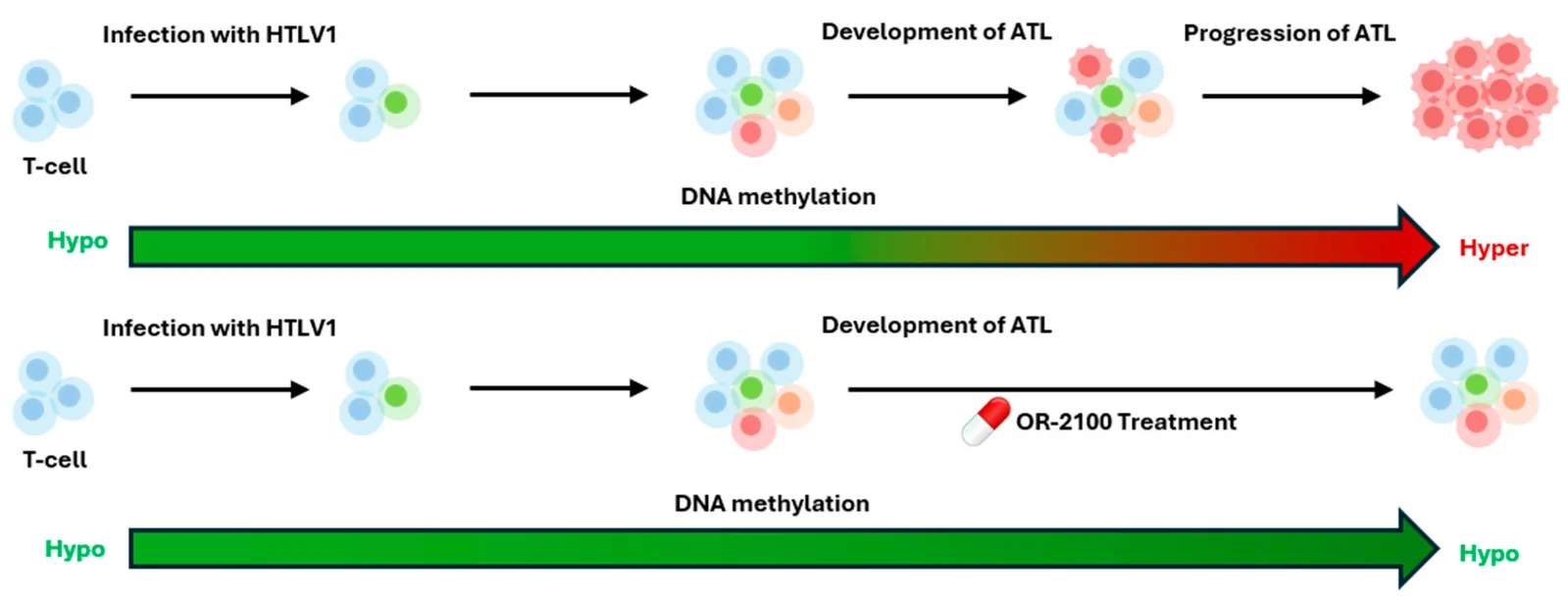
Schematic illustration of ATL progression associated with DNA hypermethylation and the therapeutic impact of OR-2100.

## Data Availability

Not applicable.
